# *APOL1* G1 genotype modifies the association between HDLC and kidney function in African Americans

**DOI:** 10.1186/s12864-015-1645-7

**Published:** 2015-05-30

**Authors:** Amy R. Bentley, Jasmin Divers, Daniel Shriner, Ayo P. Doumatey, Orlando M. Gutiérrez, Adebowale A. Adeyemo, Barry I. Freedman, Charles N. Rotimi

**Affiliations:** Center for Research on Genomics and Global Health, National Human Genome Research Institute, National Institutes of Health, Bethesda, MD USA; Department of Biostatistical Sciences, Wake Forest School of Medicine, Winston-Salem, NC USA; Division of Nephrology, University of Alabama at Birmingham, Birmingham, AL USA; Department of Internal Medicine/Nephrology, Wake Forest School of Medicine, Winston-Salem, NC USA

**Keywords:** Apolipoprotein L1, High-density lipoprotein cholesterol, African ancestry, Glomerular filtration rate

## Abstract

**Background:**

Despite evidence of an association between variants at the apolipoprotein L1 gene (*APOL1*) locus and a spectrum of related kidney diseases, underlying biological mechanisms remain unknown. An earlier preliminary study published by our group showed that an *APOL1* variant (rs73885319) modified the association between high-density lipoprotein cholesterol (HDLC) and estimated glomerular filtration rate (eGFR) in African Americans. To further understand this relationship, we evaluated the interaction in two additional large cohorts of African Americans for a total of 3,592 unrelated individuals from the Howard University Family Study (HUFS), the Natural History of *APOL1*-Associated Nephropathy Study (NHAAN), and the Atherosclerosis Risk in Communities Study (ARIC). The association between HDLC and eGFR was determined using linear mixed models, and the interaction between rs73885319 genotype and HDLC was evaluated using a multiplicative term.

**Results:**

Among individuals homozygous for the risk genotype, a strong inverse HDLC-eGFR association was observed, with a positive association in others (p for the interaction of the rs73885319 × HDLC =0.0001). The interaction was similar in HUFS and NHAAN, and attenuated in ARIC. Given that ARIC participants were older, we investigated an age effect; age was a significant modifier of the observed interaction. When older individuals were excluded, the interaction in ARIC was similar to that in the other studies.

**Conclusions:**

Based on these findings, it is clear that the relationship between HDLC and eGFR is strongly influenced by the *APOL1* rs73885319 kidney risk genotype. Moreover, the degree to which this variant modifies the association may depend on the age of the individual. More detailed physiological studies are warranted to understand how rs73885319 may affect the relationship between HDLC and eGFR in individuals with and without disease and across the lifespan.

**Electronic supplementary material:**

The online version of this article (doi:10.1186/s12864-015-1645-7) contains supplementary material, which is available to authorized users.

## Background

The apolipoprotein L1 gene (*APOL1*) has been the focus of considerable interest in recent years because of the discovery of two coding genetic variants (G1 and G2) that dramatically increase recessive risk of kidney diseases among African Americans (AAs) [1–3]. Interestingly, these variants are specific to African ancestry populations, in which they are common. It is thought that these variants rose to high frequency because they provide protection from African sleeping sickness, although it has been suggested that selection for the G1 allele may reflect a broader protective effect against pathogens [4]. While the association between *APOL1* variants and kidney disease has been confirmed for nephropathies of differing etiologies, including focal segmental glomerulosclerosis [1, 5], HIV-associated nephropathy [5], hypertension-attributed end-stage kidney disease (ESKD) [1], severe lupus nephritis [6], and chronic kidney disease (CKD) progression [7], the biological mechanism by which *APOL1* variants influence renal function has not been elucidated. Understanding the underlying relationships could have significant impact as it may suggest treatment options for those with (or without) these risk variants.

High density lipoprotein cholesterol (HDLC) has been positively associated with kidney function and inversely with CKD risk [8–11] (although there are reports of an opposite association [12–14]). The atheroprotective properties of the HDL particle may also protect glomerular cells from damage and subsequent kidney dysfunction [15–18]. In a preliminary analysis, our group has shown that the association between HDLC and estimated glomerular filtration rate (eGFR), differed depending on the genotype at rs73885319 which, along with a SNP in near-perfect LD (rs60910145), defines the *APOL1* G1 haplotype [14]. Specifically, among individuals with the GG genotype for rs73885319 (a kidney disease risk genotype), higher HDLC was associated with lower eGFR, while there was no association among those without this genotype. The initial observation was made in the Howard University Family Study (HUFS), a study of AAs designed to be representative of the general AA population living in Washington, DC. To further understand this relationship, we evaluated the *APOL1* × HDLC interaction in AA from two additional studies: the Natural History of *APOL1*-Associated Nephropathy study (NHAAN) [19] and the Atherosclerosis Risk in Communities study (ARIC) [20].

## Methods

### Participants and design

This analysis included AA individuals from the Howard University Family Study (HUFS), the Natural History of *APOL1*-Associated Nephropathy Study (NHAAN), and the Atherosclerosis Risk in Communities Study (ARIC). Briefly, HUFS is a study of AA that was designed to be representative of the general population of AA living in Washington, DC [21]. NHAAN is a study of AA first-degree relatives of patients with non-diabetic ESKD [19]. ARIC was designed as a multi-ethnic study of atherosclerosis and recruited individuals aged 45–64 years in Forsyth County, NC; Jackson, MS; the suburbs of Minneapolis, MN; and Washington County, MD [20]. This analysis only includes AAs from this study. ARIC data was accessed through the Database of Genotypes and Phenotypes (dbGaP) [22] (phs000280.v2.p1), specifically the GENEVA substudy (phs000090.v2.p1), through an approved request for controlled-access data. Included studies were approved by the Institutional Review Boards of Howard University (HUFS), Wake Forest School of Medicine (NHAAN), and, for ARIC, The University of North Carolina at Chapel Hill, Johns Hopkins University, University of Mississippi Medical Center, Wake Forest University, University of Minnesota, Brigham and Women's Hospital, and Baylor College of Medicine. Individuals with T2D or CKD (eGFR < 60 ml/min/1.73 m^2^) were excluded from analysis given dyslipidemia associated with these conditions.

### Measurements

In all studies, HDLC was determined using standard enzymatic procedures (in ARIC, dbGAP variable phv00022850.v1.p1 was selected). Serum creatinine levels were determined using a buffered kinetic Jaffé reaction without deproteinization on a COBAS Integra 400 Plus Analyzer (Roche Diagnostics, Indianapolis, IN) for HUFS. In NHAAN, serum creatinine was measured using creatinase enzymatic spectrophotometry (LabCorp, Burlington, NC; www.labcorp.com). For ARIC, serum creatinine levels (phv00080483.v1.p1) were determined using the modified kinetic Jaffé method [23] (DART Creatinine Reagent, Coulter Diagnostics, Hialeah, FL). For all studies, eGFR was calculated according to the race- and gender-specific Chronic Kidney Disease Collaboration equations [24].

### Genotyping

Genotyping at the *APOL1* locus has been previously described for both HUFS [14] and NHAAN [19]. For ARIC, imputed genotype data for rs73885319 was accessed (phg000248.v1), as well as genome-wide genotype data (phg000035.v1.p1) for the calculation of African ancestry proportion. The “best guess” genotypes based on the imputation were used in the analysis. In HUFS, population structure was assessed by principal component analysis using EIGENSOFT [25], with the first PC, which represents African ancestry proportion, retained as previously described [26]. African ancestry proportion was calculated using ancestry informative markers in NHAAN. In ARIC, African ancestry proportion was estimated using ADMIXTURE [27] with K = 2 and random markers. HapMap3 YRI and CEU samples were added to the ARIC samples to improve the estimations.

### Statistical analyses

HDLC was log-transformed in all analyses. As in the previous analysis, rs73885319 was coded recessively: those with the GG (kidney risk) genotype were compared with individuals with the AG or AA genotypes. The interaction between HDLC and rs73885319 was evaluated in a linear regression model with a multiplicative interaction term (HDLC × rs73885319, coded recessively). In each model evaluating the interaction term, terms for the main effect of HDLC and rs73885319 were included. The presented p_interaction_ is the p-value for the HDLC × rs73885319 term. The multiplicative interaction term was calculated using mean-centered variables to avoid collinearity. All models were adjusted for age, gender, BMI, genome-wide proportion African ancestry, and study along with the random effect of family (HUFS and NHAAN both included family members). All analyses were conducted using SAS 9.2 (SAS Institute, Cary, NC). Figures were produced using data from a Least Squares Means statement within the models (PROC MIXED; LSMEANS), such that the models predicted eGFR given a particular value of HDLC (evaluated at 30, 40, and 50 mg/dl) and genotype (GG or AG/AA) with all other terms set to their mean values. The points obtained from these conditions were then plotted using R (http://www.r-project.org/). While all models used log-transformed HDLC as a predictor, to produce a more easily interpretable figure, the log of clinically meaningful HDLC values were input into the LS Means statement and figure axes describe the relationship in mg/dl.

In previous analysis, an interaction between HDLC and the G2 variant (rs71785313, a 6 bp deletion) on eGFR was not observed; however, as most studies of *APOL1* evaluate the combined genotype of G1 (captured by rs73885319) and G2 as the total number of variant alleles at this locus, we also investigated the combined genotype and G2 separately, as follows. First, we modeled the interaction term as described above, but with the *APOL1* risk genotype defined as those with two copies of variant alleles for either G1 or G2 (i.e. homozygous for rs73885319 G or rs71785313 Del or compound heterozygotes; there were no individuals homozygous for the risk genotypes for both variants). For comparison, we also modeled the interaction term rs71785313 (coded recessively) × HDLC. As the G2 allele was not available in ARIC, these models only included HUFS and NHAAN participants.

## Results

Included participants are described in Table 1. The frequency of the GG genotype in HUFS was similar to what was observed among AAs in the Exome Sequencing Projects (5.1 %; http://evs.gs.washington.edu/EVS/). The frequency in NHAAN was more than twice that, consistent with ascertainment of first-degree relatives of patients with non-diabetic ESKD enriching for the genetic risk factor. In ARIC, the GG frequency was lower than expected. Although the imputation score for this variant was good (IMPUTE2 info score of 0.862), this distribution may reflect the fact that rs73885319 was imputed in ARIC (but genotyped in both HUFS and NHAAN). The distribution of eGFR was consistent with an age effect, with higher values observed in the studies with younger participants. As expected given national statistics for AAs, mean BMI for each study indicates a high degree of overweight and obesity, with substantially higher mean BMI observed among women.

**Table 1:** Participant characteristics by study

	HUFS		NHAAN		ARIC	
	Men	Women	Men	Women	Men	Women
N	486	763	220	347	690	1086
Age (yrs)	42.3 ± 12.8	41.7 ± 13.1	46.4 ± 13.7	44.9 ± 13.1	53.1 ± 5.9	52.3 ± 5.5
BMI (kg/m^2^)	28.7 ± 7.6	31.4 ± 8.9	29.3 ± 7.2	33.2 ± 9.0	27.3 ± 4.7	30.1 ± 6.3
African Ancestry (%)	79.9 ± 11.2	79.0 ± 11.6	79.5 ± 11.1	80.4 ± 9.8	83.0 ± 11.0	83.0 ± 10.9
rs73885319 GG (%)	21 (4.3 %)	31 (4.1 %)	25 (11.4 %)	34 (9.8 %)	9 (1.3 %)	27 (2.5 %)
HDLC (mg/dl)	50.5 ± 14.9	54.7 ± 14.9	48.1 ± 15.3	53.1 ± 16.8	51.6 ± 17.4	58.7 ± 17.1
rs71785313 −/− (%)	10 (2.1 %)	18 (2.4 %)	6 (2.7 %)	11 (3.2 %)	--	--
2 *APOL1* risk alleles (%)^1^	60 (12.5 %)	89 (12.1 %)	46 (20.9 %)	89 (25.7 %)	--	--
eGFR (ml/min/1.73 m^2^)	106.8 ± 18.8	107.7 ± 20.9	98.6 ± 20.9	100.9 ± 21.4	78.2 ± 12.0	78.0 ± 11.9

^1^Individuals with either rs73885319 GG or rs71785313 −/− or heterozygous for both rs73885319 and rs71785313 (no individuals were homozygous for both rs73885319 GG and rs71785313 −/−)

**Fig. 1:**
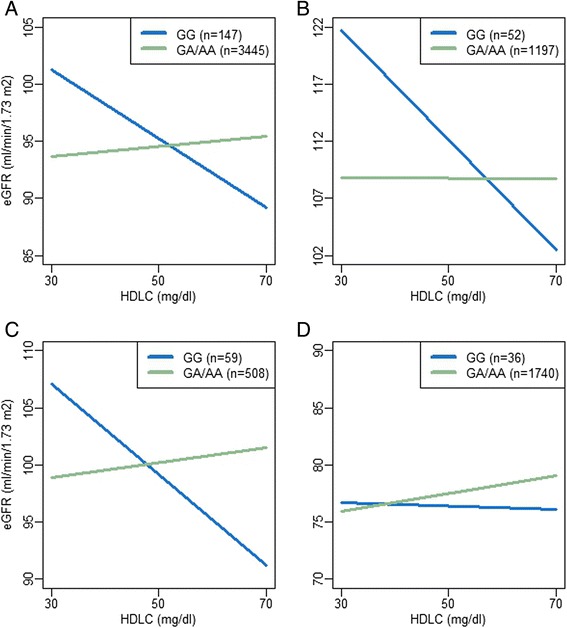
Association between eGFR and HDLC by rs73885319 genotype in African Americans. Plots from a model of eGFR as predicted by rs73885319 × HDLC, adjusted for rs73885319, HDLC, age, BMI, sex, genome-wide proportion African ancestry, study (for plot A), and the random effect of family. **a**. All African Americans; **b**. Howard University Family Study (HUFS); **c**. Natural History of APOL1-Associated Nephropathy study (NHAAN); **d**. Atherosclerosis Risk in Communities study (ARIC)

**Fig. 2:**
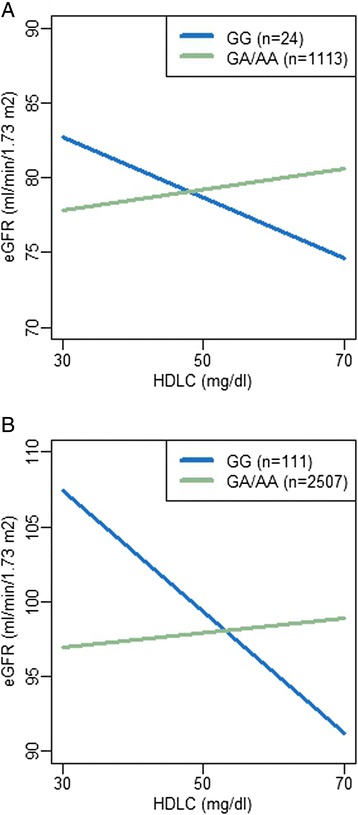
Association between eGFR and HDLC by rs73885319 genotype in African Americans < 55 years. Plots from a model of eGFR as predicted by rs73885319 × HDLC, adjusted for rs73885319, HDLC, age, BMI, sex, genome-wide proportion African ancestry, study (for plot B), and the random effect of family. **a**. Atherosclerosis Risk in Communities study (ARIC); **b**. All African Americans

The association between HDLC and eGFR varied based on genotype (p-value for HDLC × rs73885319 term [p_interaction_] = 0.0001; Fig. 1a, Table 2). Among the 147 individuals with the GG genotype, a steep inverse association between HDLC and eGFR was observed (β -0.27 per 1 mg/dl increase in HDLC, p = 0.04 in a stratified analysis). In contrast, among the 3445 individuals with the AA or AG genotype, a positive association was observed (β 0.05, p = 0.02). Notably, among those with rs73885319 AA/AG, the observed HDLC-eGFR association was similar to what we previously observed among non-African ancestry individuals [14]. When each study was considered separately (Fig. 1b-d), similar interactions were observed in HUFS (p_interaction_ = 0.005; Fig. 1b) and NHAAN (p_interaction_ = 0.006; Fig. 1c). The association in ARIC, however, was quite different (p_interaction_ = 0.6; Fig. 1d), with the slope of the association greatly attenuated among GG individuals compared to what was observed in HUFS and NHAAN. Given that participants in ARIC were older than in HUFS and NHAAN, we hypothesized that the difference in the interaction might be a function of age. We tested this hypothesis in the full sample with an interaction term for HDLC × rs73885319 × age, with age represented as a binary variable contrasting those ≥55 years with those <55 years. The 3-way interaction term for HDLC × rs73885319 × age was statistically significant (p_interaction_ = 0.02). When individuals ≥55 years were excluded, the association in ARIC was more similar to what was observed for HUFS and NHAAN (Fig. 2a); however, there were only 24 GG individuals remaining, and the interaction did not reach statistical significance (p_interaction_ = 0.17). When HUFS, NHAAN, and ARIC were jointly analyzed excluding older individuals, the interaction was similar to that in the full set, but more statistically significant (p_interaction_ = 0.00002; Table 2; Fig. 2b).

**Table 2:** Evaluation of the interaction between rs73885319 and HDLC on eGFR among African Americans by study

	rs73885319		logHDL		rs73885319 × logHDL	
	β (SE)	P-value	β (SE)	P-value	β (SE)	P-value
HUFS, NHAAN, and ARIC combined	−1.32 (1.26)	0.30	4.69 (2.08)	0.02	−37.42 (9.74)	0.0001
HUFS	0.51 (2.45)	0.83	−0.12 (4.30)	0.98	−52.12 (18.55)	0.005
NHAAN	−3.73 (2.34)	0.11	7.09 (6.11)	0.25	−50.35 (18.18)	0.006
ARIC	−1.84 (2.06)	0.37	8.03 (2.24)	0.0003	−10.30 (17.03)	0.55
ARIC (≤55 years)	−2.17 (2.59)	0.40	7.69 (2.88)	0.008	−29.78 (21.78)	0.17
HUFS, NHAAN, and ARIC combined (≤55 years)	−1.24 (1.50)	0.41	5.31 (2.54)	0.04	−49.47 (11.44)	0.00002

Results are from a model of eGFR as predicted by rs73885319 × HDLC, adjusted for rs73885319, HDLC, age, BMI, sex, genome-wide proportion of African ancestry, study (where combined), and the random effect of family

*APOL1* effects are generally described in terms of “number of risk variants,” a collapsing of variation at the G1 and G2 haplotypes. rs73885319, evaluated in this study, effectively captures the G1 haplotype, as the other G1 SNP, rs60910145, is in near-perfect LD with rs73885319 (r^2^ = 1.0 among AFR [28]). We also considered G2 separately and with G1 (number of *APOL1* risk variants) in this analysis. These analyses were limited to the HUFS and NHAAN datasets (the G2 variant was not available in ARIC). No interaction was observed with the G2 haplotype (p_interaction_ = 0.9; Table 3). When number of *APOL1* risk variants were evaluated, the interaction was weaker than was observed with rs73885319 on its own (presented here with only HUFS and NHAAN for comparison). The results from this coding are consistent with the dilution of the rs73885319 interaction by including G2.

**Table 3:** Evaluation of the interaction between APOL1 risk variants and HDLC on eGFR among African Americans^1^

	*APOL1* variant		logHDL		*APOL1* variant × logHDL	
*APOL1* Variant tested	β (SE)	P-value	β (SE)	P-value	β (SE)	P-value
rs73885319 (only)	−1.48 (1.69)	0.38	1.85 (3.53)	0.60	−44.3 (12.8)	0.0006
rs71785313 (only)	0.05 (2.58)	0.98	−0.70 (3.47)	0.84	−2.8 (22.5)	0.90
*APOL1* risk genotype (both)^2^	−1.47 (1.12)	0.19	3.02 (3.72)	0.42	−24.1 (8.7)	0.005

Results from a model of eGFR as predicted by APOL1 variant × HDLC, adjusted for variant, HDLC, age, BMI, sex, genome-wide proportion African ancestry, study, and the random effect of family. ^1^Data are presented for HUFS and NHAAN only, as rs71785313 was not available in ARIC; ^2^Individuals with either rs73885319 GG or rs71785313 −/− or heterozygous for both rs73885319 and rs71785313 (no individuals were homozygous for both rs73885319 GG and rs71785313 −/−) compared to individuals who were heterozygous for either rs73855319 or rs71785313 or homozygous for the reference allele for both

To be sure that the recessive coding of rs73885319 was appropriate, we also evaluated the addition of an interaction term for heterozygotes (rs73885319 AG × HDLC) to the model (Additional file 1). The regression coefficient (β) for the rs73885319 AG × HDLC term was neither intermediate (suggesting an additive effect) nor similar to that of the rs73885319 GG × HDLC term (suggesting a dominant effect). It was not statistically significant (p_interaction_ = 0.5) and the inclusion of this term did not affect the size or significance of the rs73885319 GG × HDLC term; thus, we feel confident that the interaction pertains only to individuals with the GG genotype, and the recessive coding best fits our data.

To evaluate the robustness of the rs73885319 × HDLC findings to analytic strategy, we conducted sensitivity analyses. First, since dyslipidemia frequently occurs jointly with T2D and CKD, individuals with these conditions were excluded to limit their influence on HDLC. When these individuals were included, the interaction remained statistically significant, though attenuated (p_interaction_ = 0.01; Additional file 2). Second, the choice of an appropriate kidney function measure can be debated. While we describe results using eGFR as estimated by the Chronic Kidney Disease Collaboration equations [24], similar interactions were observed when using eGFR calculated using the Modification of Diet in Renal Disease study equations [29] or with serum creatinine concentration (data not shown). Finally, there was no difference in rs73885319 × HDLC interaction by gender (p value for the interaction of gender × rs73885319 × HDLC = 0.56).

## Discussion

In 3,592 African American participants from three independent studies (HUFS, NHAAN, and ARIC), we demonstrated that the association between HDLC and eGFR is significantly influenced by the kidney risk genotype (GG) at rs73885319 in the *APOL1* gene. Among the 147 individuals with the GG genotype, a steep inverse association between HDLC and eGFR was observed. In contrast, among the 3445 individuals with the AA or AG genotype, HDLC was positively associated with eGFR. The mechanism underlying this *APOL1* × HDLC interaction is not clear. A potential explanation that has been recently suggested [30] is that rs73885319 directly or indirectly leads to qualitative changes in the HDL particle that render it dysfunctional in terms of its protective properties. While HDL is generally characterized as an anti-inflammatory, antioxidant particle, under certain conditions, including coronary artery disease, diabetes mellitus (types 1 and 2), metabolic syndrome, and kidney disease (reviewed in [31]), it loses some of its beneficial properties, potentially becoming pro-inflammatory and pro-oxidant. The generally protective function of the HDL particle is attributed to its role in reverse cholesterol transport as well as to the lipid and protein components of which it is composed. HDL is a heterogeneous macromolecular complex that contains more than 80 proteins and peptides, more than 200 lipid species, and several microRNAs [31]. The distribution of these components is responsive to a variety of biological changes, such as inflammation and oxidative stress [31]. During the acute phase response, for instance, HDL was shown to have reduced apoA-I levels and paraoxonase activity and increased ceruloplasmin (an acute phase reactant) [32]. HDL proteomic remodeling was observed in patients with coronary artery disease; compared to the HDL from healthy individuals, the HDL from patients had higher apoC-III and lower clustering, and stimulated endothelial pro-apoptotic pathways [33]. In the Nurses’ Health Study and the Health Professionals’ Follow-up Study, HDL proteomic differences, specifically apoC-III presence or absence, determined the direction of association between HDL and cardiovascular disease risk, with the HDL/apoC-III complex associated with increased risk [34]. With such findings, it is becoming increasingly appreciated that the measurement of HDLC, the cholesterol content of the HDL particle, is simply a proxy for particle number, and does not represent the particle’s proteome and lipidome, which may be more important in terms of disease risk. It is expected that studies evaluating the content of HDL particles in a more refined way would be informative for the interaction observed in this study.

Differences in the rs73885319 × HDLC interaction were seen by age. An older age could represent increased oxidative stress and inflammation, among other factors. While we were not able to further evaluate the underlying mechanism of this difference in these studies, one potential explanation is that aging influences the composition and/or function of the HDL particle in ways that are relevant for this interaction. Aging has been previously associated with decreased HDL-mediated reverse cholesterol transport [35], HDL antioxidant activity [36], and PON1 activity, irrespective of changes in PON1 or HDLC concentration [37]. Thus, in the context of HDL with decreased protective capacity as observed with aging, *APOL1*-influenced changes to HDL quality may be more difficult to detect.

It would be reasonable to suspect that the observed modification of the association between HDLC and eGFR by rs73885319 may be relevant for cardiovascular disease risk, as HDLC has generally been associated with improved cardiovascular health and *APOL1* kidney disease risk variants have been associated with increased CVD risk [38] (although the evidence is not consistent across studies [7, 39, 40]). It may be hypothesized that if rs73885319 contributes directly or indirectly to qualitative changes in the HDL particle that alter the relationship between HDL and kidney function, those changes might also affect the association between HDLC and CVD-related outcomes in unexpected ways. Given the uncertainty regarding the underlying biological mechanisms that this statistical interaction captures, it is difficult to speculate what may be observed.

## Conclusions

The present study confirms and extends the earlier observation that the association between HDLC and eGFR differs depending on rs73885319 genotype at the *APOL1* kidney disease locus. Among individuals with the rs73885319 GG (kidney disease risk) genotype, higher HDLC was associated with lower eGFR, while a positive association was observed among those without this genotype. In this analysis, a modulatory effect of age on the rs73885319 × HDLC interaction was observed, such that the interaction was attenuated among older individuals. Functional studies are needed to reveal the pathophysiologic mechanisms underlying this interaction.

### Availability of supporting data

All of the ARIC data used in this analysis was from the GENEVA substudy and can be accessed through dbGaP (http://www.ncbi.nlm.nih.gov/projects/gap/cgi-bin/study.cgi?study_id=phs000090.v2.p1). Participants in HUFS and NHAAN were not consented for wide data release (such as in a publicly-available database), but data can be made available for collaborative research. Additionally, summary-level statistics on these data will be provided upon request.

## Additional files

Additional file 1:
**Effect of variant coding on** ***APOL1*** **rs73885319 × HDLC interaction.** Results from a model of eGFR in HUFS, NHAAN, and ARIC individuals including terms for rs73885319 (coded as # of variant alleles), HDLC, rs73885319 AG × HDLC, rs73885319 GG × HDLC, age, BMI, sex, study, and genome-wide proportion African ancestry and a random term for family. Additional file 2:
**Association between eGFR and HDLC by rs73885319 genotype, T2D and CKD included.** Results from a model of eGFR as predicted by rs73885319 × HDLC, adjusted for rs73885319, HDLC, age, BMI, sex, genome-wide proportion African ancestry, study, and the random effect of family. In contrast to main models, no exclusion has been made for type 2 diabetes (T2D) or chronic kidney disease (CKD).

## Abbreviations

AA: African American
*APOL1*: Apolipoprotein L1
apoC-III: apolipoprotein C-III
apoA-I: apolipoprotein A-I
ARIC: Atherosclerosis risk in communities study
BMI: Body mass index
CEU: Utah residents with ancestry from Northern and Western Europe
CKD: Chronic kidney disease
dbGaP: database of Genotypes and Phenotypes
eGFR: estimated glomerular filtration rate
ESKD: End-stage kidney disease
HapMap: International HapMap (Haplotype Map) Project
HDL: High-density lipoprotein
HDLC: High-density lipoprotein cholesterol
HUFS: Howard University Family Study
NHAAN: Natural history of APOL1-associated nephropathy
PON1: Paraoxonase 1
T2D: Type 2 diabetes
YRI: Yoruba from Ibadan, Nigeria
