# Supplementary material for: APOL1 G1 genotype modifies the association between HDLC and kidney function in African Americans
Source: BMC Genomics. 2015 May 30;16(1):421. doi: 10.1186/s12864-015-1645-7 (PMC4448293; doi:10.1186/s12864-015-1645-7)
Supplement: Additional file 1: — Effect of variant coding on APOL1 rs73885319 × HDLC interaction. Results from a model of eGFR in HUFS, NHAAN, and ARIC individuals including terms for rs73885319 (coded as # of variant alleles), HDLC, rs73885319 AG × HDLC, rs73885319 GG × HDLC, age, BMI, sex, study, and genome-wide proportion African ancestry and a random term for family. [file 12864_2015_1645_MOESM1_ESM.pdf]

Effect of variant coding on *APOL1* rs73885319 × HDLC interaction[1]

Variable	β (SE)	P-value
logHDL	3.5 (4.4)	0.43
rs73885319 AG	0.05 (2.6)	0.98
rs73885319 GG	-1.8 (1.7)	0.31
rs73885319 AG × HDLC	-4.0 (6.6)	0.54
rs73885319 GG × HDLC	-46.1 (13.1)	0.0005

[1]Results are for a model of eGFR in HUFS, NHAAN, and ARIC individuals including terms for rs73885319 (coded as # of variant alleles), HDLC, rs73885319 AG × HDLC, rs73885319 GG × HDLC, age, BMI, sex, study, and genome-wide proportion African ancestry and a random term for family.
