# Supplementary material for: APOL1 G1 genotype modifies the association between HDLC and kidney function in African Americans
Source: BMC Genomics. 2015 May 30;16(1):421. doi: 10.1186/s12864-015-1645-7 (PMC4448293; doi:10.1186/s12864-015-1645-7)
Supplement: Additional file 2: — Association between eGFR and HDLC by rs73885319 genotype, T2D and CKD included. Results from a model of eGFR as predicted by rs73885319 × HDLC, adjusted for rs73885319, HDLC, age, BMI, sex, genome-wide proportion African ancestry, study, and the random effect of family. In contrast to main models, no exclusion has been made for type 2 diabetes (T2D) or chronic kidney disease (CKD). [file 12864_2015_1645_MOESM2_ESM.pdf]

Association between eGFR and HDLC by rs73885319 genotype, T2D and CKD included.

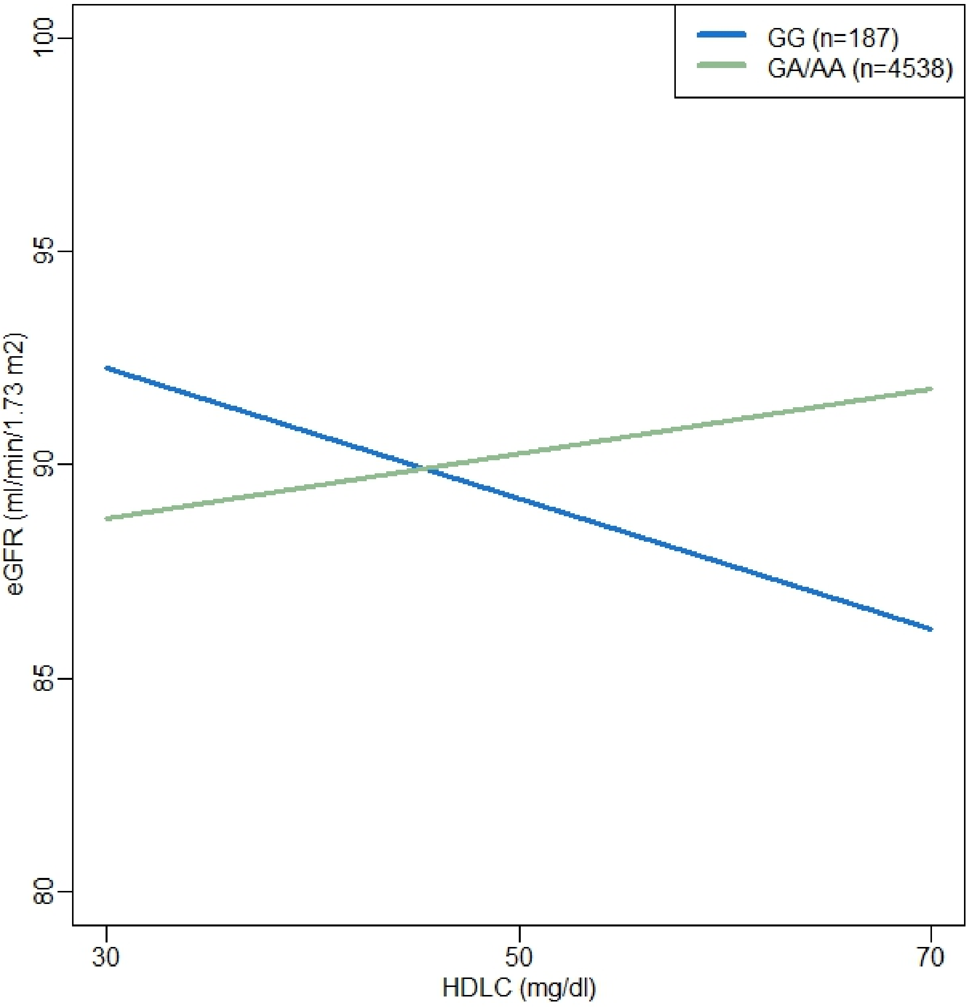

Results from a model of eGFR as predicted by rs73885319 × HDLC, adjusted for rs73885319, HDLC, age, BMI, sex, genome-wide proportion African ancestry, study, and the random effect of family. In contrast to main models, no exclusion has been made for type 2 diabetes (T2D) or chronic kidney disease (CKD).
